# Correct Sorting of Lipoproteins into the Inner and Outer Membranes of Pseudomonas aeruginosa by the Escherichia coli LolCDE Transport System

**DOI:** 10.1128/mBio.00194-19

**Published:** 2019-04-16

**Authors:** Christian Lorenz, Thomas J. Dougherty, Stephen Lory

**Affiliations:** a Department of Microbiology, Blavatnik Institute, Harvard Medical School, Boston, Massachusetts, USA; Emory University School of Medicine

**Keywords:** Lol pathway, *Pseudomonas aeruginosa*, lipoproteins, outer membrane

## Abstract

Biogenesis of the outer membrane of Gram-negative bacteria depends on dedicated macromolecular transport systems. The LolABCDE proteins make up the machinery for lipoprotein trafficking from the inner membrane (IM) across the periplasm to the outer membrane (OM). The Lol apparatus is additionally responsible for differentiating OM lipoproteins from those for the IM. In *Enterobacteriaceae*, a default sorting mechanism has been proposed whereby an aspartic acid at position +2 of the mature lipoproteins prevents Lol recognition and leads to their IM retention. In other bacteria, the conservation of sequences immediately following the acylated cysteine is variable. Here we show that in Pseudomonas aeruginosa, the three essential Lol proteins (LolCDE) can be replaced with those from Escherichia coli. The P. aeruginosa lipoproteins MexA, OprM, PscJ, and FlgH, with different sequences at their N termini, were correctly sorted by either the E. coli or P. aeruginosa LolCDE. We further demonstrate that an inhibitor of E. coli LolCDE is active against P. aeruginosa only when expressing the E. coli orthologues. Our work shows that Lol proteins recognize a wide range of signals, consisting of an acylated cysteine and a specific conformation of the adjacent domain, determining IM retention or transport to the OM.

## Abstract (precis)

Gram-negative bacteria build their outer membranes (OM) from components that are initially located in the inner membrane (IM). A fraction of lipoproteins is transferred to the OM by the transport machinery consisting of LolABCDE proteins. Our work demonstrates that the LolCDE complexes of the transport pathways of Escherichia coli and Pseudomonas aeruginosa are interchangeable, with the E. coli orthologues correctly sorting the P. aeruginosa lipoproteins while retaining their sensitivity to a small-molecule inhibitor. These findings question the nature of IM retention signals, identified in E. coli as aspartate at position +2 of mature lipoproteins. We propose an alternative model for the sorting of IM and OM lipoproteins based on their relative affinities for the IM and the ability of the promiscuous sorting machinery to deliver lipoproteins to their functional sites in the OM.

## INTRODUCTION

The cell envelope of Gram-negative bacteria contains a set of proteins that are tethered to either the inner membrane (IM) or the outer membrane (OM) via fatty acids attached to their amino-terminal cysteines (1). These lipoproteins not only contribute to the integrity of the cell envelope but are also components of various bacterial nanomachines, including the flagellar apparatus, the peptidoglycan biosynthesis machinery, and various extracellular transport systems for proteins, lipopolysaccharide, and antibiotics (2–5). Moreover, in many pathogenic organisms, lipoproteins represent a group of highly proinflammatory molecules and play an important role in host responses during infection (6, 7).

In Gram-negative bacteria, a substantial fraction of the lipoproteins is found in the OM. A dedicated lipoprotein localization machinery decodes the information within the mature, fully acylated mature lipoproteins and directs their targeting to the OM, which includes extraction from the IM, transport across the periplasm, and incorporation in the OM in a functional form (8–10). In gammaproteobacteria, the lipoprotein transport pathway consists of a LolCDE ATP-binding cassette transporter responsible for the release of the OM-targeted lipoproteins from the IM and directing them into a complex with the periplasmic molecular chaperone LolA. The final step in the lipoprotein biogenesis is their transfer from LolA into the OM; this process is facilitated by the OM lipoprotein LolB (3–5).

Since in Gram-negative bacteria both membranes of the cell envelope contain lipoproteins that function specifically at these locations, the LolCDE also has a sorting activity, i.e., it can differentiate between lipoproteins that remain in the IM and those that are targeted to the OM. A short stretch of N-terminal amino acid residues contains what is referred to as a “Lol avoidance” or “IM retention” signal; in its absence, the lipoproteins are directed to the Lol OM transport pathway (11, 12). The key feature of this signal is the lack of recognition by LolCDE or potential interference with the transfer to the periplasmic chaperone, LolA. In Escherichia coli, this signal is the highly conserved aspartic acid at the +2 position of the mature lipoprotein, usually followed by aspartate, glutamate, or glutamine residues. The positioning of the aspartate, and the absence of basic residues immediately adjacent to it, is referred to as a strong Lol avoidance signal; its location within the membrane containing basic phosphatidyl ethanolamine is not recognized by LolCDE, and therefore these lipoproteins remain in the IM (13).

Identification of a large number of bacterial lipoproteins from whole-genome sequences showed that the Lol avoidance signal, based on the conservation of aspartic acid at the +2 position, is less common outside enterobacterial species. In Pseudomonas aeruginosa, where the aspartic acid is rarely found at the +2 position, Lol avoidance appears to be determined by a combination of amino acids at the +3 and +4 positions (14, 15). Specificities of the Lol machinery have been studied through heterologous expression of lipoproteins. For example, MexA, the IM lipoprotein component of the P. aeruginosa efflux system, contains a glycine residue at position +2, and when expressed in E. coli, it was found in the OM fraction when its localization was assessed using sucrose gradient centrifugation. Substituting aspartic acid for the same glycine did not affect the localization of MexA G2D in P. aeruginosa but resulted in colocalization with an OM protein in E. coli, suggesting that the basis of strain specificity is the coevolution of the Lol machinery with Lol avoidance signals in distinct bacterial species (15).

The evolutionary conservation of the aspartate residue at position +2 and its mutagenesis causing mislocalization have been interpreted as evidence that this particular amino acid represents a critical determinant for lipoproteins to avoid extraction from the IM by LolCDE and transfer to LolA for OM targeting (11, 16). However, in contrast to the above findings, several studies have suggested that *Pseudomonas* IM lipoproteins lacking the aspartate IM retention signal are recognized and properly localized by the E. coli Lol apparatus (17–19). We therefore investigated whether the LolCDE components of the P. aeruginosa lipoprotein transport machinery can be replaced by their orthologues from E. coli and whether these can correctly localize lipoproteins into the IM and OM compartments. We demonstrate that LolCDE from E. coli can restore the viability of P. aeruginosa *ΔlolCDE* and that it can correctly localize four lipoproteins in the cell envelope in their functional forms. We additionally show that a small-molecule inhibitor of the E. coli Lol transport can exert the same toxic effect in P. aeruginosa only in strains expressing the E. coli orthologues. This observation suggests that this molecule functions by binding to unique sites on the E. coli LolC or LolE and that activity against divergent Lol systems for a broad-spectrum drug will require a design approach based on the structure of the inhibitor and its protein target.

## RESULTS

### Replacement of P. aeruginosa LolCDE with the LolCDE orthologues.

**FIG 1:**
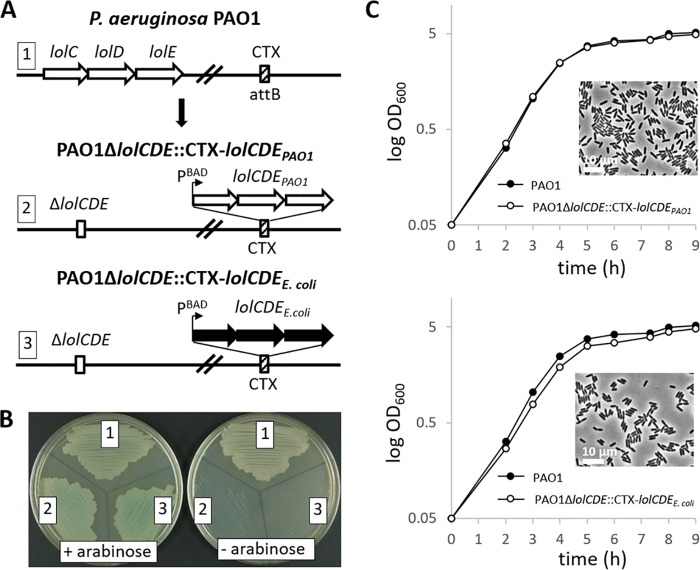
LolCDE replacement in P. aeruginosa PAO1. (A) Schematic cloning strategy for LolCDE replacement. The *lolCDE* genes from P. aeruginosa PAO1 (white arrows) or E. coli MG1655 (black arrows) were inserted into the PAO1 genome at the CTX phage attachment site (attB) under the control of an arabinose-inducible promoter (P_BAD_). Subsequently, the native PAO1 *lolCDE* genes were deleted from their genomic locus by homologous recombination of flanking regions. (B) Arabinose-dependent growth of LolCDE replacement strains. Constructs correspond to the numbering shown in panel A. Streaks of strains PAO1 (1), PAO1 Δ*lolCDE*::CTX-*lolCDE*_PAO1_ (2), and PAO1 Δ*lolCDE*::CTX-*lolCDE_E. coli_* (3) on LB agar plates containing (+) or lacking (–) l-arabinose are shown. (C) Growth curves of PAO1 wild type (black circles) and PAO1 Δl*olCDE*::CTX-*lolCDE*_PAO1_ (white circles, upper panel) or PAO1 Δ*lolCDE*::CTX-*lolCDE_E. coli_* (white circles, lower panel). Cells were diluted from overnight cultures and grown in LB medium with 0.5% l-arabinose at 37°C with shaking. The optical density at a wavelength of 600 nM (OD_600_) was monitored over the course of 9 h. Microscope images of PAO1 Δ*lolCDE*::CTX-*lolCDE*_PAO1_ (upper panel) and PAO1 Δ*lolCDE*::CTX-*lolCDE_E. coli_* (lower panel) cells from mid-log phase were taken with a Nikon Eclipse Ti-E microscope.

In order to compare the specificities of the E. coli and P. aeruginosa Lol pathways during the early steps in lipoprotein transport, we replaced the *lolCDE* genes of P. aeruginosa with those from E. coli (9, 14). We created P. aeruginosa PAO1 strains with deleted native *lolCDE* genes into which had been inserted either the E. coli *lolCDE* or P. aeruginosa *lolCDE* genes (under the control of the arabinose-inducible P_BAD_ promoter) in the CTX phage attachment site. These constructs are shown schematically in Fig. 1A. We confirmed the essentiality of *lolCDE* gene products by demonstrating that the viability of P. aeruginosa Δ*lolCDE* carrying the E. coli or P. aeruginosa *lolCDE* genes, PAO1 Δ*lolCDE*::CTX-*lolCDE_E. coli_* and PAO1 Δ*lolCDE*::CTX-*lolCDE*_PAO1_*_,_* respectively, depends on the presence of the l-arabinose inducer in the growth medium (Fig. 1B). Moreover, the growth kinetics of induced PAO1 Δ*lolCDE*::CTX-*lolCDE*_PAO1_ and PAO1 Δ*lolCDE*::CTX-*lolCDE_E. coli_* are nearly identical to those of the PAO1 wild-type strain (Fig. 1C). The expression of either LolCDE did not result in any apparent difference in bacterial morphology when bacteria were examined by phase-contrast microscopy (Fig. 1C, insets). The ability of the E. coli LolCDE complex to complement the essential early lipoprotein transport functions (extraction of lipoproteins from IM and transfer to LolA) suggests that the adherence to the Lol avoidance signals, at least for the essential P. aeruginosa lipoproteins, is not absolute.

### E. coli LolCDE complex directs correct functional localization of P. aeruginosa lipoproteins.

Using PAO1 Δ*lolCDE*::CTX-*lolCDE_E. coli_*, we examined the ability of the heterologous machinery to sort P. aeruginosa lipoproteins that function in the IM (MexA and PscJ,) and two that are targeted to the OM (OprM and FlgH). It is noteworthy that none contain the *E. coli* Lol avoidance aspartic acid residue signal at the +2 position. Further examples in Table S2 in the supplemental material find only 3 of 17 P. aeruginosa IM lipoproteins with aspartate at position +2.

**FIG 2:**
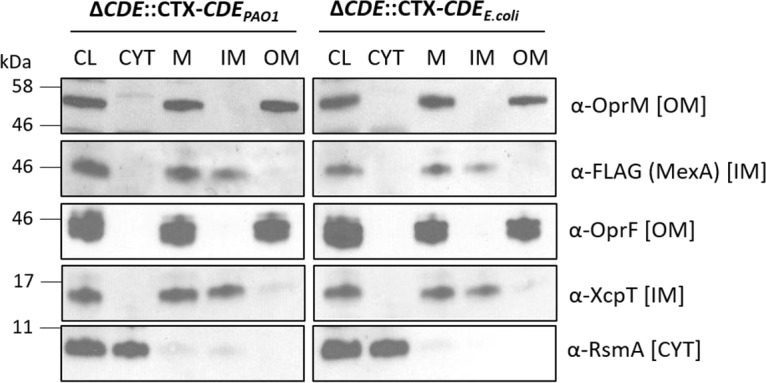
Subcellular localization of lipoproteins MexA and OprM in P. aeruginosa PAO1 LolCDE replacement strains. Cell fractionation studies were done with strains PAO1 Δ*lolCDE*::CTX-*lolCDE*_PAO1_ and PAO1 Δ*lolCDE*::CTX-*lolCDE_E. coli_* carrying pMMB67EH-*mexA*-FLAG. Immunoblot analyses assess the subcellular localization (CL, cleared lysate; CYT, cytoplasm; M, total membranes; IM, inner membrane; OM, outer membrane) using antibodies against the FLAG epitope (MexA), OprM and OprF (OM proteins), XcpT (IM protein), and RsmA (cytoplasmic protein).

**(i) Sorting of P. aeruginosa MexA and OprM lipoproteins.** To address the sorting of lipoproteins in the P. aeruginosa strain with the LolCDE complex from E. coli, we analyzed the function of the MexAB-OprM efflux pump (20) and performed cell fractionation studies of its lipoprotein components. Functional efflux requires the lipoprotein OprM, the antibiotic conduit in the OM, and the MexA membrane fusion protein anchored in the IM. To determine the localization of the lipoproteins, we used a detergent-based fractionation protocol to separate IM and OM proteins, followed by immunoblotting of the same extracts using anti-OprM rabbit polyclonal antibodies, while anti-FLAG antibodies were used to detect MexA-FLAG (a MexA-FLAG hybrid protein with the FLAG epitope fused to its C terminus, expressed from pMMB67EH-*mexA*-FLAG). Antibodies against OprF, XcpT, and RsmA were used as controls for OM, IM, and cytoplasmic protein localization, respectively. Figure 2 shows the fractionation analyses of lysates of PAO1 Δ*lolCDE*::CTX-*lolCDE_E. coli_* and PAO1 Δ*lolCDE*::CTX-*lolCDE*_PAO1_, each carrying pMMB67EH-*mexA*-FLAG. There were no major differences in localization of OprM and MexA, regardless of the source of the LolCDE, further demonstrating that the proteins from E. coli were able to recognize and target these two P. aeruginosa proteins to their correct location in the cell envelope.

To test the functionality of the P. aeruginosa MexAB-OprM efflux pump whose lipoprotein components were sorted by the E. coli LolCDE complex, we compared the antibiotic susceptibilities of strains PAO1 Δ*lolCDE*::CTX-*lolCDE_E. coli_* and PAO1 Δ*lolCDE*::CTX-*lolCDE*_PAO1_. The MICs of erythromycin, cefepime, chloramphenicol, and ciprofloxacin, known substrates of the MexAB-OprM efflux pump, were comparable between PAO1 strains expressing *lolCDE_E. coli_* or *lolCDE*_PAO1_. A minor reduction in antibiotic efflux efficiency (less than 2- to 3-fold) (Table 1) is seen where lipoproteins are localized using the E. coli LolCDE complex (in PAO1 Δ*lolCDE*::CTX-*lolCDE_E. coli_*). However, both strains were significantly more resistant (with 10- to 40-fold higher MICs) than a P. aeruginosa mutant lacking the MexAB-OprM efflux pump (Δ*mexAB-oprM*). This result shows that not only are the MexA and OprM lipoproteins correctly localized into their respective IM and OM locations using either the P. aeruginosa or E. coli LolCDE, but along with MexB, they also form a fully functional system for antibiotic efflux.

**TABLE 1:** MICs of MexAB-OprM efflux pump substrates in P. aeruginosa PAO1 LolCDE replacement strains and PAO1 *mexAB-oprM* deletion strain

Drug	MIC (µg/ml) of *P. aeruginosa* strain		
	PAO1 Δ*lolCDE*::CTX-*CDE*_PAO1_	PAO1 Δ*lolCDE*::CTX-*CDE_E. coli_*	PAO1 Δ*mexAB-oprM*
Erythromycin	48	32	3
Cefepime	1	1	0.094
Chloramphenicol	16	6	0.38
Ciprofloxacin	0.125	0.064	0.006

**FIG 3:**
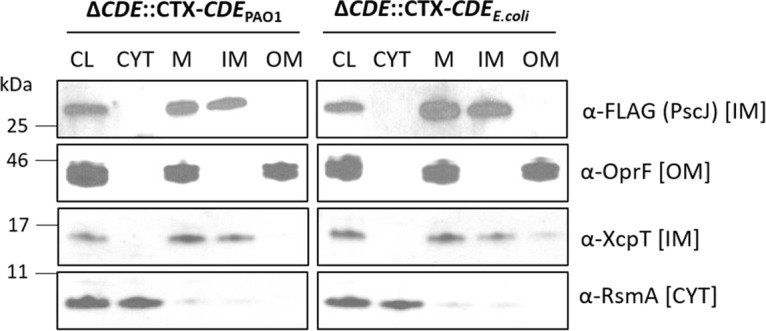
Subcellular localization of lipoprotein PscJ in P. aeruginosa PAO1 LolCDE replacement strains. Cell fractionation studies were done with strains PAO1 Δ*lolCDE*::CTX-*lolCDE*_PAO1_ and PAO1 Δ*lolCDE*::CTX-*lolCDE_E. coli_* carrying pMMB67EH-*pscJ*-FLAG. Immunoblot analyses assess the subcellular localization (CL, cleared lysate; CYT, cytoplasm; M, total membranes; IM, inner membrane; OM, outer membrane) using antibodies against the FLAG epitope (PscJ), OprF (OM protein), XcpT (IM protein), and RsmA (cytoplasmic protein).

**(ii) Sorting of P. aeruginosa PscJ lipoprotein component of the type III secretion system.** Another lipoprotein involved in transport across the Gram-negative cell envelope is PscJ, an IM lipoprotein component of the P. aeruginosa type III secretion system (T3SS) (21). Fractionation of the P. aeruginosa IM and OM compartments from PAO1 Δ*lolCDE*::CTX-*lolCDE_E. coli_* and PAO1 Δ*lolCDE*::CTX-*lolCDE*_PAO1_, expressing PscJ with a C-terminal FLAG epitope from plasmid pMMB67EH-*pscJ*-FLAG, showed that PscJ is retained in the IM in both strains (Fig. 3).

**FIG 4:**
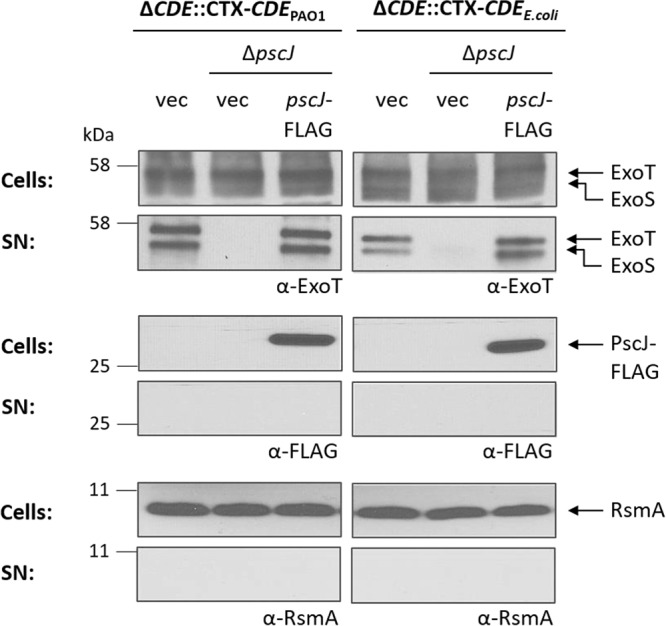
Function of the type III secretion system in P. aeruginosa PAO1 LolCDE replacement strains. Strains PAO1 Δ*lolCDE*::CTX-*lolCDE*_PAO1_, PAO1 Δ*lolCDE*::CTX-*lolCDE_E. coli_*, and *pscJ* deletion derivatives carrying pMMB67EH vector (vec) or pMMB67EH-*pscJ*-FLAG (*pscJ*-FLAG) as indicated were incubated in type III secretion-inducing low-calcium medium. Immunoblot analyses of cells and secreted proteins in the culture supernatants (SN) were done using antibodies against ExoT, the FLAG epitope (PscJ), and RsmA. The ExoT antibody recognizes both ExoT (upper band) and ExoS (lower band) (24, 41). The immunoblots were probed with an antibody against the cytoplasmic RsmA protein as a lysis control.

We next assessed the functionality of the T3SS by determining the extent of secretion of two proteins, ExoS and ExoT, utilizing the T3SS of P. aeruginosa strains with the LolCDE complex from E. coli and P. aeruginosa. In these strains, we created an additional mutation by deleting the chromosomal *pscJ* gene, allowing us to monitor the levels of PscJ from a plasmid-borne gene (22, 23). Cell-associated and secreted proteins were analyzed by Western immunoblotting, using rabbit polyclonal antibodies raised against ExoT (Fig. 4). Since ExoS and ExoT share 62% sequence identity, anti-ExoT also recognizes ExoS and the same antibody can be used to monitor the secretion of both of the toxins (24). The analysis of normalized whole-cell lysates and supernatant fractions shows equivalent secretion levels of ExoT and ExoS from PAO1 Δ*pscJ* Δ*lolCDE*::*lolCDE*_PAO1_ and PAO1 Δ*pscJ* Δ*lolCDE*::*lolCDE_E. coli_* in cells expressing comparable levels of PscJ-FLAG. In spite of lacking the Asp at position +2, PscJ avoids recognition by E. coli LolCDE and is retained, in a fully functional form, in the IM.

**FIG 5:**
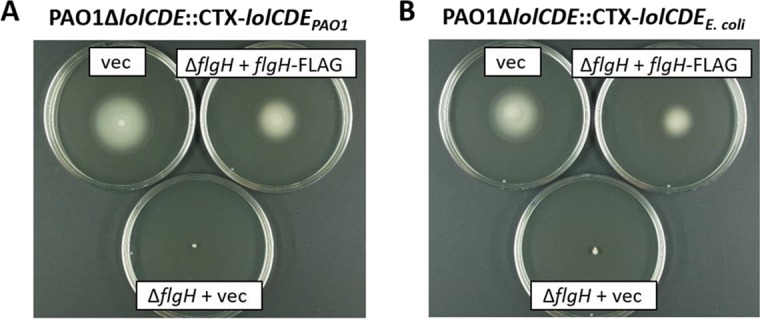
Swimming motility of P. aeruginosa PAO1 LolCDE replacement strains. Motility phenotypes of strains PAO1 Δ*lolCDE*::CTX-*lolCDE*_PAO1_ (A), PAO1 Δ*lolCDE*::CTX-*lolCDE_E. coli_* (B), and *flgH* deletion derivatives carrying pMMB67EH (vec) or pMMB67EH-*flgH*-FLAG (*flgH*-FLAG) as indicated on soft LB agar (0.3%) plates.

**FIG 6:**
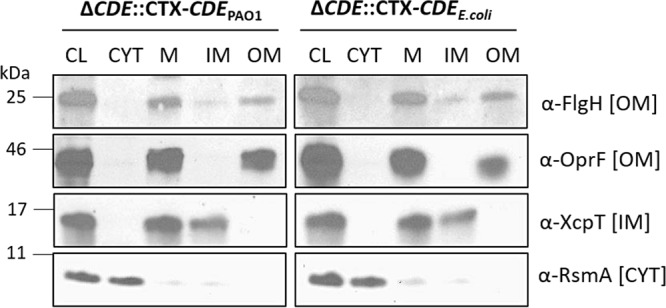
Subcellular localization of lipoprotein FlgH in P. aeruginosa PAO1 LolCDE replacement strains. Cell fractionation studies with strains PAO1 Δ*lolCDE*::CTX-*lolCDE*_PAO1_ and PAO1 Δ*lolCDE*::CTX-*lolCDE_E. coli_*. Immunoblot analyses assessing the subcellular localization (CL, cleared lysate; CYT, cytoplasm; M, total membranes; IM, inner membrane; OM, outer membrane) were done using antibodies against FlgH, OprF (OM protein), XcpT (IM protein), and RsmA (cytoplasmic protein).

**(iii) E. coli LolCDE complex targets P. aeruginosa lipoprotein FlgH into the OM.** The flagella are multiprotein structures that function as the organelles of bacterial motility. The sole lipoprotein component of the flagellar basal body is FlgH, a protein assembled into a ring structure in the OM (25). We compared the functional assemblies of flagella in strains with *lolCDE_E. coli_* and *lolCDE*_PAO1_ by monitoring their swimming motility on soft agar plates. As seen in Fig. 5, PAO1 Δ*lolCDE*::CTX-*lolCDE_E. coli_* and PAO1 Δ*lolCDE*::CTX-*lolCDE*_PAO1_ displayed comparable swimming phenotypes, and these were dependent on FlgH, as the deletion of its gene in both strain backgrounds results in nonmotile bacteria (Fig. 5). This phenotype can be complemented by expressing FlgH with a C-terminal FLAG epitope from plasmid pMMB67EH-*flgH*-FLAG in the *flgH* mutant strains. In addition to the motility assay, fractionation of the membranes into inner and outer membranes demonstrated that FlgH was correctly localized to the outer membrane with both the P. aeruginosa and E. coli *lolCDE* constructs (Fig. 6). Therefore, strain PAO1 Δ*lolCDE*::CTX-*lolCDE_E. coli_* is capable of correctly transporting FlgH to the outer membrane, leading to its incorporation into the basal body and resulting in functional flagella.

### Reducing the barrier function of the P. aeruginosa OM.

A pyrazole-containing small-molecule inhibitor of LolCDE has been previously identified by Nayar et al. (26). This compound (referred to as compound 2) was active against wild-type E. coli and was more active against an efflux-deficient isogenic mutant, with MICs of 8 and 0.125 µg/ml, respectively. The compound showed no activity against P. aeruginosa, raising the possibility that it fails to penetrate its OM. Alternatively, the compound could display a strict specificity toward the E. coli LolCDE and simply not interact with the P. aeruginosa orthologues. We took advantage of the P. aeruginosa strain expressing E. coli LolCDE to examine the potential target spectrum of this inhibitor of lipoprotein transport.

**FIG 7:**
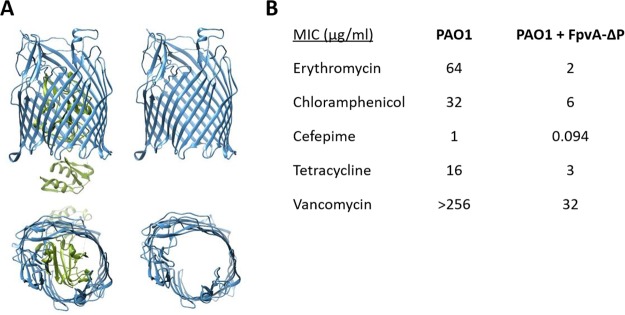
Reducing the P. aeruginosa OM permeability barrier by expressing the plugless FpvA pore. (A) Structures of FpvA parent, with plug in green (left), and engineered FvpA-ΔP version lacking the central plug (amino acids 48 to 276) (right). Upper structures, side views; lower structures, top views. (B) MICs of selected antibiotics against P. aeruginosa PAO1 and PAO1 expressing plugless FpvA pore from plasmid pMMB67EH-*fpvA*-*ΔP* induced with 500 µM IPTG.

We have engineered a P. aeruginosa strain expressing a modified P. aeruginosa pyoverdine transporter, FpvA. Previously, Scott et al. and Krishnamoorty et al. (27, 28) have shown that the expression of E. coli siderophore uptake channel FepA or FhuA, lacking the central plug domain, significantly reduced the barrier function of the OM and sensitized E. coli to killing by poorly penetrating antibiotics. We engineered a similar construct by deleting the N-terminal plug domain of P. aeruginosa FpvA (referred to as FpvA-ΔP). The structure of this protein (Fig. 7A) shows a predicted open channel of ca. 25 Å. We then assessed the antibiotic susceptibility of P. aeruginosa expressing this mutant FpvA. Compared to wild-type P. aeruginosa PAO1, strain PAO1 *fpv-ΔP* showed enhanced sensitivity to five selected antibiotics that differed in their intracellular targets, molecular sizes, and physicochemical properties (Fig. 7B). The most significant enhancement of antibiotic susceptibility was seen with erythromycin (32-fold). Vancomycin, a large glycopeptide, was inactive against wild-type PAO1, but the expression of the FpvA lacking the plug domain rendered the PAO1 *fpv-ΔP* strain sensitive to this antibiotic (MIC = 32 µg/ml). Therefore, similar to the findings with modified FhuA, the expression of Fpv-ΔP leads to an increase in OM permeability in P. aeruginosa and should facilitate passage of relatively small compounds, such as compound 2A, across the OM (28).

### Compound inhibition of E. coli LolCDE in P. aeruginosa.

**FIG 8:**
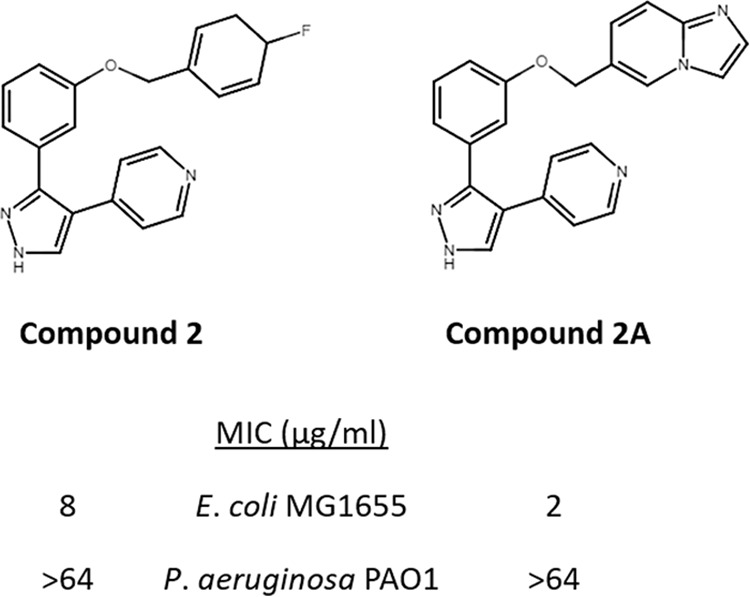
LolCDE inhibitor compound 2 and derivative compound 2A. Chemical structures (top) and MICs (bottom) against E. coli MG1655 and P. aeruginosa PAO1 are shown.

We examined the ability of the small-molecule inhibitor of LolCDE to exert its lethal activity against P. aeruginosa strains with different origins of the LolCDE complex. We used a modified version of compound 2 (compound 2A) (Fig. 8) that has been previously shown to be more potent against E. coli than the parental compound (29). We confirmed that compound 2A is 4-fold more potent than compound 2 in its antibacterial activity (Fig. 8). Neither compound 2 nor compound 2A showed activity against wild-type P. aeruginosa PAO1. In order to determine whether the lack of activity of compound 2A in P. aeruginosa was due to poor permeability, efflux, or a lack of interaction with the P. aeruginosa LolCDE orthologues, we tested its killing activity in P. aeruginosa with LolCDE*_E. coli_* or LolCDE_PAO1_. We also assessed the contributions of the MexAB-OprM efflux pump and the alteration in OM permeability using the FpvA-ΔP construct to the killing activity of compound 2A. As shown in Table 2, the viability of P. aeruginosa PAO1 Δ*lolCDE*::CTX-*lolCDE*_PAO1_ is not affected by compound 2A, even in the absence of the MexAB-OprM efflux pump or the expression of FpvA-ΔP or in strains lacking the efflux pump and also expressing the FpvA-ΔP pore to compromise the outer membrane permeability barrier. In contrast, the P. aeruginosa Δ*lolCDE*::CTX-*lolCDE_E. coli_* strain becomes more susceptible to compound 2A when either the MexAB-OprM efflux pump is lacking or the permeability of its OM is increased by the expression of the FpvA-ΔP protein; in each case, these strains show an MIC of 16 µg/ml. A further 4-fold reduction in susceptibility to 4 µg/ml was seen when the *mexAB-oprM* deletion and FpvA-ΔP overexpression were combined in the P. aeruginosa Δ*lolCDE*::CTX-*lolCDE_E. coli_* background. These results show that whereas the LolCDE proteins of E. coli and P. aeruginosa are functionally interchangeable, the compound 2A inhibitor likely makes contacts with residues that are found in the binding regions in E. coli LolCDE that differ in the P. aeruginosa orthologues.

**TABLE 2:** MICs of compound 2A against strains PAO1 Δ*lolCDE*::CTX-*lolCDE*_PAO1_ and PAO1 Δ*lolCDE*::CTX-*lolCDE_E. coli_* and derivatives^*a*^

Strain modification	MIC (µg/ml) of compound 2A for:	
	PAO1 Δ*lolCDE*::CTX-*CDE*_PAO1_	PAO1 Δ*lolCDE*::CTX-*CDE_E. coli_*
None	>64	>64
Δ*mexAB-oprM*	>64	16
+ FpvA-ΔP	>64	16
Δ*mexAB-oprM* + FpvA-ΔP	>64	4

a The *Pseudomonas* PAO1 strains with the E. coli and P. aeruginosa LolCDE systems were grown in the presence of arabinose. FpvA-ΔP expression from plasmid pMMB67EH-*fpvA*-*ΔP* was induced with 200 µM IPTG.

## DISCUSSION

In this study, we examined the specificities of sorting machineries for a group of lipoproteins that significantly differ at their mature N termini, which likely functions as a sorting signal determining IM retention or OM transport. In E. coli and other *Enterobacteriaceae*, an aspartic acid residue at the +2 position is found in almost all lipoproteins (with occasionally, an aspartic acid at +3) that are retained in the IM (11). This residue is considered a Lol avoidance signal, as it has been shown to interfere with the recognition by the Lol transport apparatus, specifically by LolCDE (11), and the lipoproteins remain in the IM. In many other Gram-negative bacterial species, lipoproteins that are localized and function in the IM lack a strong preference for any specific amino acid within the sequence immediately following the acylated N-terminal cysteine. The amino acid sequences at this N-terminal region are not completely random; for example, in P. aeruginosa, lysine at the +3 position or serine at +4 are more frequent than others and were suggested to function as the Lol retention signals in this organism (15, 30). However, the list of 40 likely IM lipoproteins of P. aeruginosa shows that lysine and serine are found at positions +3 and +4 in only 5 and 8 instances, respectively, while the majority of these lipoproteins contain variable sequences with a preference for acidic residues for the five amino acids following the N-terminal cysteine (30, 31). Table S1 in the supplemental material includes a compiled list of annotated inner and outer membrane lipoproteins showing four amino acids following the cysteine.

10.1128/mBio.00194-19.2 TABLE S1 Annotated inner membrane lipoproteins compiled from the work of Remans et al. (https://doi.org/10.1099/mic.0.040659-0). Annotated functions were updated where necessary at http://pseudomonas.com/; only functionally annotated proteins are included. Four-amino-acid region following cysteine of mature protein is indicated. Annotated outer membrane lipoproteins are examples, not a comprehensive list. Cys + 4 region of mature lipoproteins is indicated. Download Table S1, DOCX file, 0.06 MB. Copyright © 2019 Lorenz et al. 2019 Lorenz et al. This content is distributed under the terms of the Creative Commons Attribution 4.0 International license.

10.1128/mBio.00194-19.3 TABLE S2 Primers employed. Restriction sites are underlined, and epitope tag-encoding sequences are in italics. Download Table S2, DOCX file, 0.09 MB. Copyright © 2019 Lorenz et al. 2019 Lorenz et al. This content is distributed under the terms of the Creative Commons Attribution 4.0 International license.

Another indication that lipoproteins lacking an aspartate residue at position +2 are able to avoid the targeting function of the Lol machinery came from studies evaluating the activities of two P. aeruginosa efflux pumps, MexAB-OprM and MexCD-OprJ, in E. coli (17–19). The function of these so-called tripartite pumps in effluxing antibiotics requires the assembly of the components into a complex consisting of an ABC transporter (MexB and MexD) in the IM linked to the OM component (OprM and OprJ) through an IM lipoprotein (MexA and MexC). When expressed in E. coli, the bacteria displayed a multidrug resistance phenotype, indicating that the pumps were correctly assembled in the cell envelope and that they were functional. Moreover, the substrate antibiotic specificities of the heterologous MexAB-OprM and MexCD-OprJ in E. coli were similar to those seen in P. aeruginosa (17). Neither MexA nor MexC contain the aspartate residue at the +2 position of the mature lipoprotein, yet both were retained in the IM and avoid OM transport by the E. coli Lol machinery, suggesting that lipoproteins do not necessarily need to adhere to the Asp position +2 Lol avoidance paradigm to remain and function in the IM.

We have shown that the E. coli LolCDE complex can function in P. aeruginosa and correctly localize essential lipoproteins, as well as specific ones lacking an aspartate at the +2 position, namely MexA, OprM, PscJ, and FlgH. This demonstrates that the early steps of sorting of the lipoproteins, including their avoidance of OM trafficking, do not depend on a specific amino acid signal. These results are in line with a previous report on additional lipoprotein retention sequences identified in *Pseudomonas* (31). Moreover, our results also demonstrate that the heterologously expressed LolCDE appears to correctly interact with other components of the pathway (LolA and LolB) in P. aeruginosa. Previously, Grabowicz and Silhavy (32) have suggested that LolA and LolB function primarily as chaperones facilitating the hydrophobic lipidated proteins to reach the OM, preventing their misfolding into inactive or potentially toxic forms; these two Lol proteins may be dispensable when lipoprotein levels are reduced and the Cpx stress response is activated.

Our data raise further questions about the sorting mechanisms that allow IM retention of lipoproteins functioning in this membrane (33). The early steps of lipoprotein modification, the processing of the signal peptide by Lsp, and the addition of fatty acids by Lgt and Lnt are conserved for all lipoproteins; therefore the presence of an acylated cysteine alone is insufficient to determine their IM or OM localization (3, 34). Based on the poor conservation of sequences adjacent to the N-terminal cysteines in most bacterial species (30), and the demonstrated interchangeability of the LolCDE proteins between E. coli and P. aeruginosa, we propose a general model for lipoprotein sorting, whereby their retention in the IM is a function of the N-terminal domain’s ability to assume a conformation that facilitates strong interaction within the IM, likely with phospholipids. This may include homo-oligomerization or formation of complexes with other IM proteins. In contrast, those lipoproteins with an amino acid composition weakly associated with the IM bilayer are extracted by the Lol apparatus and transported to the OM. Given that the amino acid composition of the N-terminal region of mature lipoproteins is variable (30, 31), it is not surprising that the Lol machinery is promiscuous and can recognize a wide range of proteins that need to be transported and/or maintained in a conformation to prevent their misfolding in the periplasm. Moreover, probing this region by creating substitution mutations is likely to be uninformative, since it would be dependent on the deviation from the native sequence; a consequence is that structural features of the domain would be expected to change. Different amino acid substitutions that alter the affinity of the lipoprotein for the IM could be structurally altered in unpredictable ways, leading to either no effect or mislocalization. It is therefore difficult to assign a precise signal function to a domain that can be readily perturbed by substitutions for amino acids not found in the native proteins. However, it remains unclear why the IM lipoproteins of *Enterobacteriaceae*, unlike other Gram-negative bacteria, show such a strong preference for aspartic acid at the +2 position (3, 4, 11).

We have also used the P. aeruginosa strain expressing heterologous LolCDE from E. coli to evaluate strain specificity of a LolCDE inhibitor, compound 2A, a more potent derivative in E. coli of compound 2 (29). This compound shows no activity against P. aeruginosa expressing endogenous LolCDE, a mutant lacking the efflux pump MexAB-OprM, or when the OM for this strain was modified by coexpression of the modified FpvA-ΔP protein. However, the expression of *E. coli* LolCDE in P. aeruginosa resulted in its killing by compound 2A, with enhanced activity due to permeabilization of the OM with FpvA-ΔP and a further increase in susceptibility when the *mexAB-oprM* genes were deleted. Thus, the compound inhibits only P. aeruginosa expressing the E. coli LolCDE version. Some of the key amino acids in LolC and LolE identified in resistant E. coli mutants as important for inhibitor activity differ from those present at equivalent positions in alignments with *Pseudomonas* LolCDE (Fig. S1). Hence, the P. aeruginosa LolCDE is refractory to inhibition by the compound very likely because of a lack of key compound binding sites targeted in the E. coli orthologues. Further structural studies and interrogation of the E. coli LolCDE-inhibitor complex could shed light on key interactions with the compound 2 inhibitors, and structure-based design using the refractory P. aeruginosa LolCDE could yield an inhibitor of lipoprotein transport with a broader spectrum of activity (35).

10.1128/mBio.00194-19.1 FIG S1 Alignments of LolC and LolE of E. coli and P. aeruginosa. Arrows indicate critical base changes between the E. coli and *Pseudomonas* protein sequences. In E. coli, mutational amino acid changes at any one of these positions in either LolC or LolE results in high-level resistance to compound 2. The differences in amino acids at these key positions account for the lack of activity of the compound in *Pseudomonas* possessing the native P. aeruginosa *lolCDE* genes. Download FIG S1, DOCX file, 0.1 MB. Copyright © 2019 Lorenz et al. 2019 Lorenz et al. This content is distributed under the terms of the Creative Commons Attribution 4.0 International license.

## MATERIALS AND METHODS

### Bacterial strains and culture conditions.

P. aeruginosa and E. coli were routinely cultured in Luria-Bertani (LB) medium at 37°C with shaking at 300 rpm. Strain genotypes and plasmids are listed in Table 3. Antibiotics were used at the following concentrations: tetracycline (Tc) at 30 μg/ml, carbenicillin (Cb) at 75 μg/ml, and gentamicin (Gm) at 75 μg/ml for P. aeruginosa, and tetracycline at 10 μg/ml, ampicillin (Amp) at 100 μg/ml, gentamicin at 15 μg/ml, and kanamycin (Km) at 50 μg/ml for E. coli.

**TABLE 3:** Strains and plasmids used in this study

Strain or plasmid	Genotype or relevant properties	Reference
Strains		
P. aeruginosa		
PAO1	Wild-type strain	42
PAO1 Δ*mexAB-oprM*	PAO1 with unmarked *mexAB-oprM* deletion	This study
PAO1 Δ*lolCDE*::*lolCDE*_PAO1_	*lolCDE* deletion strain with PAO1-*lolCDE* inserted into CTX phage attachment site (Tc^r^)	This study
PAO1 Δ*lolCDE*::*lolCDE_E. coli_*	*lolCDE* deletion strain with E. coli *lolCDE* inserted into CTX phage attachment site (Tc^r^)	This study
PAO1 Δ*pscJ* Δ*lolCDE*::*lolCDE*_PAO1_	PAO1 Δ*lolCDE*::*lolCDE*_PAO1_ with *pscJ* deletion	This study
PAO1 Δ*pscJ* Δ*lolCDE*::*lolCDE_E. coli_*	PAO1 Δ*lolCDE*::*lolCDE_E. coli_* with *pscJ* deletion	This study
PAO1 Δ*mexAB-oprM* Δ*lolCDE*::*lolCDE*_PAO1_	PAO1 Δ*lolCDE*::*lolCDE*_PAO1_ with *mexAB-oprM* deletion	This study
PAO1 Δ*mexAB-oprM* Δ*lolCDE*::l*olCDE_E. coli_*	PAO1 Δ*lolCDE*::*lolCDE_E. coli_* with *mexAB-oprM* deletion	This study
PAO1 Δ*flgH* Δ*lolCDE*::*lolCDE*_PAO1_	PAO1 Δ*lolCDE*::*lolCDE*_PAO1_ with *flgH* deletion	This study
PAO1 Δ*flgH* Δ*lolCDE*::*lolCDE_E. coli_*	PAO1 Δ*lolCDE*::*lolCDE_E. coli_* with *flgH* deletion	This study
E. coli		
MG1655	F^–^ lambda^–^ *rph-1*	43
DH5α	F^–^ φ80*lacZ*ΔM15 Δ(*lacZYA*-*argF*)*U169 deoR recA1 endA1 hsdR17* (r_K_^−^ m_K_^−^) *phoA supE44* λ^–^ *thi-1 gyrA96 relA1*	Invitrogen
Plasmids		
pSW196	Site-specific integrative plasmid, P_BAD_ promotor, *attB* (Tc^r^)	44
pSW196-*lolCDE*_PAO1_	pSW196 carrying PAO1 *lolCDE*	This study
pSW196-*lolCDE_E. coli_*	pSW196 carrying E. coli *lolCDE*	This study
pMMB67EH	IncQ, RSF1010, *lacI*^q^ P*tac*, expression vector (Amp^r^)	45
pMMB67EH-*mexA*-FLAG	*mexA*-FLAG expression construct	This study
pMMB67EH-*pscJ*-FLAG	*pscJ*-FLAG expression construct	This study
pMMB67EH-*flgH*-FLAG	*flgH*-FLAG expression construct	This study
pMMB67EH-*fpvA*-*ΔP*	Plugless *fpvA* expression construct	This study
pEXG2	Allelic exchange vector (Gm^r^)	46
pEXG2Δ*mexAB-oprM*	*mexAB-oprM* deletion construct	This study
pEXG2Δ*pscJ*	*pscJ* deletion construct	This study
pEXG2Δ*flgH*	*flgH* deletion construct	This study
pRK2013	Helper plasmid with conjugative properties (Km^r^)	47

### Primers.

Primers for PCR, used in all constructions, are listed in Table S2 in the supplemental material.

### LolCDE replacement.

The *lolCDE* genes from P. aeruginosa or E. coli were cloned into the EcoRI/SpeI sites of pSW196 under the control of an arabinose-inducible P_BAD_ promoter. pSW196-*lolCDE*_PAO1_ and pSW196-*lolCDE*_E. coli_ were conjugated into PAO1 using triparental mating. Tetracycline-resistant transconjugants were checked for genomic insertion of the *lolCDE* genes at the CTX site by PCR and sequencing.

### P. aeruginosa *lolCDE* deletion.

Following the introduction of either P. aeruginosa or E. coli *lolCDE* genes into the CTX site, for deletion of the *lolCDE* genes at their original genome locus, ∼500 bp of upstream and downstream regions flanking the native *Pseudomonas lolCDE* genes were cloned into pEXG2 (36). The resulting plasmid, pEXG2Δ*lolCDE*, was conjugated into the PAO1 strains with *lolCDE* insertions at the CTX site. Transconjugants with deletion of the genomic *lolCDE* alleles were selected on medium containing 6% sucrose and 0.5% l-arabinose. Resolved strains were tested for gentamicin sensitivity, and *lolCDE* deletion was confirmed by sequencing using primers in the upstream and downstream genes flanking the native *lolCDE* operon. Deletions of *mexAB-oprM*, *pscJ,* and *flgH* were done in a similar fashion by utilizing pEXG2.

### Cell fractionation.

P. aeruginosa strains carrying either pMMB67EH-*mexA*-FLAG or pMMB67EH-*pscJ*-FLAG were grown at 37°C in 100 ml LB medium with 0.5% l-arabinose, tetracycline (30 µg/ml), and carbenicillin (75 µg/ml) to an optical density at 600 (OD_600_) of 0.6, induced with 200 µM IPTG (isopropyl-β-d-thiogalactopyranoside), and incubated for an additional 2 h. Cells were pelleted by centrifugation (4,700 × *g* for 15 min at 4°C), resuspended in 2 ml of resuspension buffer (20 mM Tris-HCl, pH 7.5, 100 mM NaCl, 1 mM EDTA), and lysed using glass beads (acid washed, <106 µm; Sigma) and vortexing. The lysate was centrifuged at 18,000 × *g* for 10 min at 4°C to remove glass beads, intact cells, and cell debris. Cytoplasmic and membrane fractions of 1 ml of cleared lysate were separated by ultracentrifugation (Beckman Optima TLX ultracentrifuge, rotor TLA120.2) for 45 min at 200,000 × *g* at 4°C. The membrane pellet was resuspended in 1 ml of inner membrane solubilization buffer (20 mM Tris-HCl, pH 7.5, 0.2% sodium lauroyl sarcosinate) and incubated on ice for 30 min (37). Inner and outer membranes were separated by ultracentrifugation (as described above), and the outer membrane pellet was resuspended in 1 ml of outer membrane resuspension buffer (20 mM Tris-HCl, pH 7.5). After the addition of 2× Laemmli buffer, the samples were boiled for 5 min and the proteins were separated by SDS-PAGE and then transferred to nitrocellulose membranes for detection by specific antibodies using enhanced chemiluminescence.

### MIC determination.

The MICs (38) of strains with either P. aeruginosa or E. coli *lolCDE* genes against compound 2A for strains carrying pMMB67EH or pMMB67EH-*fpvA*-*ΔP* were determined in microtiter plates (LB medium with 0.5% l-arabinose and 75 µg/ml carbenicillin with 5 × 10^5^ CFU/ml). The MICs of erythromycin, chloramphenicol, cefepime, tetracycline, and ciprofloxacin were determined using Etest strips (bioMérieux Inc.) on LB agar plates with 0.5% l-arabinose (and 75 µg/ml carbenicillin for strains carrying pMMB67EH or pMMB67EH-*fpvA*-*ΔP*) plated with 10^6^ CFU/ml.

### Type III secretion assay.

P. aeruginosa strains carrying pMMB67EH or pMMB67EH-*pscJ*-FLAG were grown from an OD_600_ of 0.1 in LB medium with 0.5% l-arabinose, tetracycline (30 µg/ml), and carbenicillin (75 µg/ml), 200 µM IPTG, 10 mM EGTA, and 5 mM MgCl_2_ for 6 h at 37°C. Bacterial densities were determined by optical density measurements at 600 nm. Cells were pelleted by centrifugation at 18,000 × *g* for 10 min at 4°C. Proteins in the supernatant were precipitated with trichloroacetic acid and washed with ethanol. Proteins were resuspended in Laemmli buffer according to culture density, separated by SDS-PAGE, and transferred to nitrocellulose membranes for detection by specific antibodies using enhanced chemiluminescence.

### Motility assay.

Motility assays were conducted using LB medium containing 0.3% agar, 30 µg/ml tetracycline, 75 µg/ml carbenicillin, 0.5% l-arabinose, and 200 µM IPTG. P. aeruginosa strains carrying pMMB67EH or pMMB67EH-*flgH*-FLAG were tested for motility by inoculating each strain with a needle into the center of a plate containing this medium. The plates were incubated at 37°C for 16 h, after which each strain was scored for its ability to spread beyond the point of inoculation (39).

### Generation of FpvA lacking the central plug domain.

The *fpvA* gene was PCR amplified from PAO1 in two sections (N and C termini, leaving out the sequence encoding the plug [bp 48 to 276]) with a short overlap for Gibson assembly (New England Biolabs HiFi DNA assembly) (40). The resulting *fpvA*-*ΔP* (plug deletion) fragment was cloned into the EcoRI/XmaI site of pMMB67EH and confirmed by DNA sequencing.

10.1128/mBio.00194-19.4 TEXT S1 Supplemental references. Download Text S1, DOCX file, 0.01 MB. Copyright © 2019 Lorenz et al. 2019 Lorenz et al. This content is distributed under the terms of the Creative Commons Attribution 4.0 International license.
