# Supplementary material for: Correct Sorting of Lipoproteins into the Inner and Outer Membranes of Pseudomonas aeruginosa by the Escherichia coli LolCDE Transport System
Source: mBio. 2019 Apr 16;10(2):e00194-19. doi: 10.1128/mBio.00194-19 (PMC6469965; doi:10.1128/mBio.00194-19)
Supplement: TABLE S1 [file mBio.00194-19-st001.docx]

Supplemental Information

Correct sorting of lipoproteins into the inner and outer membranes of *Pseudomonas aeruginosa* by the *Escherichia coli* LolCDE lipoprotein system

Table S1.

*Pseudomonas aeruginosa* inner membrane lipoprotein examples

Protein Function Cys 4+

PA0425 MexA efflux MFP  CGKSE

PA4599 MexC efflux MFP  CGPAE

PA2493 MexE efflux MFP CGKAP

PA1723 PscJ  Type III secretion CKVEL

PA0156 TriA MFP CGAEP

PA0157 TriB MFP CGDEP

PA0867 MliC lysozyme inhibitor CGGSD

PA1222 MltA lytic transglycosylase CDDGK

PA1812 MltD lytic transglycosylase CQGSG

PA2321 GntK gluconate kinase CGKSV

PA3262 PpiC peptidyl-prolyl isomerase CDSQT

PA3396 NosL NO reductase CDASR

PA3623 NlpD amidase activator CSSSP

PA4367 BifA phosphodiesterase CAQIV

PA4370 IcmP metalloproteinase CGDDK

PA4444 MltB lytic transglycosylase CSSEP

PA5041 PilP  Type IV pilus CGGGS.

Table S1 (con’t)

*Pseudomonas aeruginosa* outer membrane lipoprotein examples

Protein Function Cys 4+

PA0427 OprM efflux CSLIP

PA4597 OprJ efflux CSMAP

PA1238 OpmJ efflux CANRN

PA4208 OpmD efflux CSVGP

PA3623 NlpD amidase activator CSSSP

PA1083 FlgH flagella CVNPP

PA3805 PilF pilin CVTSG

PA4668 LolB lipoprotein transport CAGLT

PA3988 LptE LPS CGFQL

PA3062 PelC polysaccharide CSSFT
