# Supplementary material for: Correct Sorting of Lipoproteins into the Inner and Outer Membranes of Pseudomonas aeruginosa by the Escherichia coli LolCDE Transport System
Source: mBio. 2019 Apr 16;10(2):e00194-19. doi: 10.1128/mBio.00194-19 (PMC6469965; doi:10.1128/mBio.00194-19)
Supplement: TABLE S2 [file mBio.00194-19-st002.docx]

Supplemental Information

Correct sorting of lipoproteins into the inner and outer membranes of *Pseudomonas aeruginosa* by the *Escherichia coli* LolCDE lipoprotein system

Table S2.

| **Primer name** | **Primer sequence** |
| --- | --- |
| PA-*lolCDE*-pSW196 *Eco*RI F | CGTTTTTTTGGGCTAGCGAATTCCAGCTAAGGGCGACATGTTC |
| PA-*lolCDE*-pSW196 *Spe*I R | TACTATACTAGTTCACTCGTAGCGCAGCGACTCGGC |
| EC-*lolCDE*-pSW196 *Eco*RI F | CGTTTTTTTGGGCTAGCGAATTCCAGCAACCAGACGGATTTCATGTAC |
| EC-*lolCDE*-pSW196 *Spe*I R | TACTATACTAGTTTACTGGCCGCTAAGGACTCGCGC |
| 5’ *lolCDE*-pEXG2 *Hin*dIII F | TATCAAGCTTTTCTGGAGGATATGCCGGGCCAGCAACTG |
| 5’ *lolCDE*-pEXG2 *Xba*I R | ATTCTAGATTAGGACAGGGGTCTGAACATGTCGCCCTTAG |
| 3’ *lolCDE*-pEXG2 *Xba*I F | ATTCTAGACTACGAGTGAGCGAGAAGGCCTGC |
| 3’ *lolCDE*-pEXG2 *Eco*RI R | TGGAATTCCGTCGCCTGTTCAAGCFCTACATGC |
| 5’ *mexAB-oprM*-pEXG2 *Hin*dIII F | TACTATAAGCTTTCTGGATGCGCGTCCGCACATGC |
| 5’ *mexAB-oprM*-pEXG2 *Xba*I R | TACTATTCTAGACAGGGTCACCGTCTGCGCTTCCAG |
| 3’ *mexAB-oprM*-pEXG2 *Xba*I F | TACTATTCTAGATCGCTGTTCACCGCGCAGCAGCAA |
| 3’ *mexAB-oprM*-pEXG2 *Sac*I R | TACTATGAGCTCGGCCAGGGCAAGGCGAAGAAACCG |
| 5’ *pscJ*-pEXG2 *Hin*dIII F | TACTATAAGCTTCCAGCAGCAAACCTTTCTCCTCCAG |
| 5’ *pscJ*-pEXG2 *Xba*I R | TACTATTCTAGACGCCATGCGCGACAGACCTTTCAC |
| 3’ *pscJ*-pEXG2 *Xba*I F | TACTATTCTAGACTGGCGCAGTTCTTCTGGCACCGG |
| 3’ *pscJ*-pEXG2 *Sac*I R | TACTATGAGCTCGCGCAGGCGCCGCGAGAAACCGCA |
| 5’ *flgH*-pEXG2 *Hin*dIII F | TACTATAAGCTTCCGTGTCCTACACCCGCGACGGC |
| 5’ *flgH*-pEXG2 *Xba*I R | TACTATTCTAGAGATGCCAAGCAGGGAGACGATCAT |
| 3’ *flgH*-pEXG2 *Xba*I F | TACTATTCTAGAGACCGCTTCTTCCTCAGCCCGCTG |
| 3’ *flgH*-pEXG2 *Sac*I R | TACTATGAGCTCGTTGACGGTGATCTTCGAGCCGTC |
| *mexA*-pMMB *Xba*I F | TACTATTCTAGATTCGCTCATGAGGACAACGCTATG |
| *mexA*-FLAG-pMMB *Hin*dIII R | TACTATAAGCTTTCA*CTTATCGTCGTCATCCTTGTAATC*GCCCTTGCTGTCGGTTTTCG |
| *pscJ*-pMMB *Eco*RI F | TACTATGAATTCCGAAACCCTGATGAAGACCCAATG |
| *pscJ*-FLAG-pMMB *Hin*dIII R | TACTATAAGCTTTCA*CTTATCGTCGTCATCCTTGTAATC*GCCCCGTTGCCGGTGCCAGA |
| *flgH*-pMMB *Eco*RI F | TACTATGAATTCTACGCCGCGAGGTAGCTTGATATG |
| *flgH*-FLAG-pMMB *Hin*dIII R | TACTATAAGCTTTCA*CTTATCGTCGTCATCCTTGTAATC*GAACGGCCACAGCGGGCTG |
| *fpvA* front end *Eco*RI F | CAGAATTCCAACCTAAGAAGAGCAATCACCCATGCCAGC |
| *fpvA* front end R | CGACATGGCCCTTGAACTCGACTTCCTGC |
| *fpvA* rear end F | GCAGGAAGTCGAGTTCAAGGGCCATGTCG |
| *fpvA* rear end *Xma*I R | TCTCCCCGGGTCAGAAGTCCCAGCGAGTGCTGAACATCAGG |
