# Supplementary material for: Correct Sorting of Lipoproteins into the Inner and Outer Membranes of Pseudomonas aeruginosa by the Escherichia coli LolCDE Transport System
Source: mBio. 2019 Apr 16;10(2):e00194-19. doi: 10.1128/mBio.00194-19 (PMC6469965; doi:10.1128/mBio.00194-19)

Supplemental Material

Correct sorting of lipoproteins into the inner and outer membranes of *Pseudomonas aeruginosa* by the *Escherichia coli* LolCDE lipoprotein system

Figure S1.

LolC 38.9 % identity between *E. coli* and *P. aeruginosa*


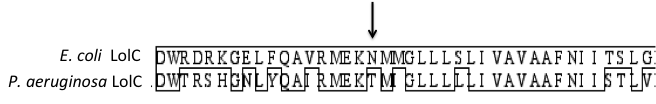


LolE 35.6% identity between *E. coli* and *P. aeruginosa*


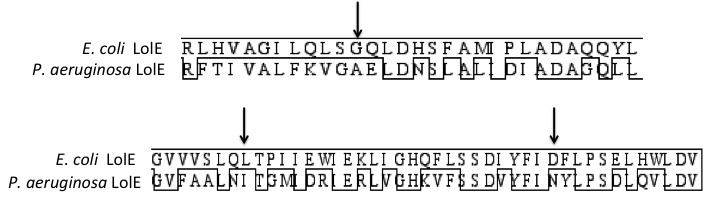

Supplement: FIG S1 [file mBio.00194-19-sf001.docx]
