# Supplementary material for: Correct Sorting of Lipoproteins into the Inner and Outer Membranes of Pseudomonas aeruginosa by the Escherichia coli LolCDE Transport System
Source: mBio. 2019 Apr 16;10(2):e00194-19. doi: 10.1128/mBio.00194-19 (PMC6469965; doi:10.1128/mBio.00194-19)
Supplement: TEXT S1 [file mBio.00194-19-s0001.docx]

**Supplement References**

1. Remans K, Vercammen K, Bodilis J, Cornelis P. 2010. Genome-wide analysis and literature-based survey of lipoproteins in Pseudomonas aeruginosa. Microbiology. 2010 156(9):2597-2607. <https://dx.doi.org/10.1099/mic.0.040659-0>

2. Nayar AS, Dougherty TJ, Ferguson KE, Granger BA, McWilliams L, Stacey C, Leach LJ, Narita S-I, Tokuda H, Miller AA, Brown DG, McLeod SM. 2015. Novel antibacterial targets and compounds revealed by a high-throughput cell wall reporter assay. J Bacteriol 197(10):1726-1734. <https://doi.org/10.1128/JB.02552-14>

3. McLeod SM, Fleming PR, MacCormack K, McLaughlin RE, Whiteaker JD, Narita S, Mori M, Tokuda H, Miller AA. 2015. Small-molecule inhibitors of gram-negative lipoprotein trafficking discovered by phenotypic screening. J Bacteriol 197(6):1075-1082. <https://doi.org/10.1128/JB.02352-14>

4. Lorenz C, Dougherty TJ, Lory S. 2016. Transcriptional responses of *Escherichia coli* to a small-molecule inhibitor of LolCDE, an essential component of the lipoprotein transport pathway. J Bacteriol 98(23):3162-3175. <https://doi.org/> 10.1128/JB.00502-16
